# Early experiences from one of the first treatment programs for chronic hepatitis B in sub-Saharan Africa

**DOI:** 10.1186/s12879-017-2549-8

**Published:** 2017-06-19

**Authors:** Hanna Aberra, Hailemichael Desalegn, Nega Berhe, Girmay Medhin, Kathrine Stene-Johansen, Svein Gunnar Gundersen, Asgeir Johannessen

**Affiliations:** 1 Medical Department, St. Paul’s Hospital Millennium Medical College, Addis Ababa, Ethiopia; 2 Aklilu Lemma Institute of Pathobiology, Addis Ababa University, Addis Ababa, Ethiopia; 3 Centre for Imported and Tropical Diseases, Oslo University Hospital, Ullevål, Box 4956 Nydalen, 0424 Oslo, PO Norway; 4 Department of Molecular Biology, Norwegian Institute of Public Health, Oslo, Norway; 5 Research Unit, Sørlandet Hospital HF, Kristiansand, Norway; 6 Department of Global Development and Planning, University of Agder, Kristiansand, Norway

**Keywords:** Hepatitis B virus, Antiviral therapy, Resource-limited settings, Africa

## Abstract

**Background:**

Treatment for chronic hepatitis B (CHB) is virtually absent in sub-Saharan Africa. Here we present early experiences from a pilot program for treatment of CHB in Ethiopia.

**Methods:**

Adults (≥18 years) with CHB were included in a cohort study at St. Paul’s Hospital Millennium Medical College, Addis Ababa, from February 2015. The baseline assessment included liver function tests, viral markers and transient elastography (Fibroscan 402, Echosense, France). Logistic regression models were used to identify predictors of fibrosis. Tenofovir disoproxil fumarate (TDF) was initiated based on the European Association for the Study of the Liver (EASL) criteria, with some modifications. The initial 300 patients underwent a more comprehensive evaluation and are presented here.

**Results:**

One-hundred-and-thirty-eight patients (46.0%) were women and median age was 30 years (interquartile range 26–40). Co-infections were rare: four patients (1.3%) were anti-HCV positive, 11 (3.7%) were anti-HDV positive, whereas 5 (1.7%) had HIV-infection. The majority were hepatitis B e-antigen (HBeAg) negative (*n* = 262; 90.7%) and had a normal (≤40 U/L) alanine aminotransferase (ALT) (*n* = 245; 83.1%). Of 268 patients with a valid Fibroscan result, 79 (29.5%) had significant fibrosis (>7.9 kPa). Independent predictors of fibrosis were male sex, age > 35 years and viral load >20,000 IU/ml. In total, 74 patients (24.7%) started TDF therapy, of whom 46 (62.2%) had cirrhosis.

**Conclusions:**

The majority were HBeAg negative and had normal ALT. However, one quarter of the patients were in need of antiviral treatment, underscoring the need to scale up CHB treatment on the African continent.

**Trial registration:**

NCT02344498 (ClinicalTrials.gov identifier). Registered 16 January 2015.

## Background

One third of the world’s population have serological evidence of past or present infection with hepatitis B virus (HBV), while 240 million people are chronic hepatitis B surface antigen (HBsAg) carriers. There are wide geographical variations, with the highest prevalence found in East Asia and Sub-Saharan Africa. Chronic hepatitis B (CHB) has a variable natural history, ranging from an inactive carrier state with an excellent long-term prognosis, to progressive liver fibrosis which may lead to the development of cirrhosis and hepatocellular carcinoma (HCC). Of adults with CHB, approximately 20–30% will develop these complications, and an estimated 686,000 people die prematurely due to HBV every year. The natural course depends on several factors, such as HBV viral load and genotype, co-infection with other viruses including hepatitis C virus (HCV), hepatitis D virus (HDV) or human immunodeficiency virus (HIV), and other host factors such as alcohol abuse and obesity [1–3].

Antiviral agents active against HBV have been shown to suppress HBV replication, prevent progression to cirrhosis, and reduce the risk of HCC and liver-related death [4–6]. International liver societies have issued guidelines for the treatment of CHB [2, 7], and a number of studies have demonstrated the safety and efficacy of antiviral treatment of HBV in high-income countries [4, 5, 8, 9]. These experiences, however, are not necessarily translatable to an African setting, where epidemiological, environmental and socioeconomic factors are different, which in turn might impact the natural history, disease progression and risk of cirrhosis and HCC [10].

In 2015, the World Health Organization (WHO) issued guidelines for the prevention, care and treatment for persons with CHB, with an emphasis on resource-limited settings [1], but still little is known about treatment indications and outcomes in sub-Saharan Africa. Only a few African countries have developed national treatment plans for CHB, and although tenofovir disoproxil fumarate (TDF) is widely available in HIV programs throughout the continent, access to antiviral therapy for CHB mono-infected individuals is severely restricted. In 2016, the WHO launched the Global Health Sector Strategy of Viral Hepatitis, with the aim of reducing deaths due to viral hepatitis by 65% within 2030 [3]; however, achieving this aim will require concerted action from both international stakeholders and the countries affected. Currently, only 9% of HBV-infected individuals have been tested, and of those diagnosed only 8% were on treatment in 2015 [11]. Hence, it is imperative to improve access to hepatitis B treatment in Africa, and real-life experiences from African treatment cohorts are urgently needed to direct implementation, policy change and local treatment guidelines.

Ethiopia is one of the most populous nations in Africa with a population size of close to 100 million. The estimated life expectancy is 65 years, and the major health problems of the country are preventable communicable diseases [12]. The sero-prevalence of HBsAg is estimated to be 7.4%, and more than 60% of chronic liver disease and up to 80% of HCC are due to hepatitis B and C infections [13]. Although HBsAg screening is widely available in the country, access to CHB treatment has been absent up to now, except through the “black market”. In 2015, we set up a pilot program for treatment and care of CHB in Ethiopia, using a simplified protocol, aiming to evaluate the implementation of modern antiviral treatment in a resource-limited setting. For scientific purposes, the initial 300 patients underwent comprehensive characterization at inclusion. Early experiences from the program are presented here.

## Methods

### Study setting and participants

A treatment program was established in February 2015 at St. Paul’s Hospital Millennium Medical College, which is located in the capital city Addis Ababa. It is a tertiary hospital delivering medical services to patients referred from all over the country. This was the first center offering CHB treatment in the country, and health care providers at other hospitals and clinics were informed about the new services provided. Hence, HBsAg positive individuals were referred both from hospital departments based on clinical liver disease, and from blood banks and antenatal care clinics based on a positive screening test.

Consenting adults (≥18 years of age) with CHB were eligible for enrolment in the cohort study, in the absence of HCC and other terminal illnesses. CHB was defined as a persistent positive HBsAg for more than 6 months [1]. In patients without a previous HBsAg test, or if the first positive HBsAg test was less than 6 months ago, HBsAg testing was repeated in order to distinguish from acute hepatitis B. Patients with HIV co-infection were transferred to the nearest HIV care and treatment center.

Although this CHB treatment center was set up as a research program with external funding, the long-term sustainability will be ensured through the National Hepatitis Program. The Ethiopian Ministry of Health, as one of the first in sub-Saharan Africa, recently issued national guidelines for the prevention and control of viral hepatitis [14], and since 2016, antiviral therapy has been available to patients with CHB at selected hospitals and clinics in the country.

Ethical clearance was obtained from the Regional Committee for Medical and Health Research Ethics in Norway and the National Research Ethics Review Committee in Ethiopia, as well as the pertinent institutional ethical review boards. Written informed consent was obtained from all study participants.

### Data collection

All patients were interviewed and examined using a standardized form. Baseline data included sociodemographic information, pregnancy/breastfeeding status, and previous use of antiviral agents and other medications. Past medical history included previous liver diseases, previous hepatitis serology, and ingestion of alcohol, khat (a psychostimulant plant native to the Horn of Africa and the Arabian peninsula) and other substances. Harmful alcohol use was defined as more than 30 g/day for men and 20 g/day for women [15], and khat abuse was defined as current use of khat, regardless of the quantity. Physical examination included liver stigmata, such as spider angiomas, jaundice and ascites.

### Laboratory analyses

Blood samples were collected at inclusion and thereafter 3-monthly, and tested locally for hematology, biochemistry and standard serology. HBsAg was detected using a WHO approved rapid diagnostic test (Determine, Alere Inc., USA). Routine laboratory investigations were carried out using the following kits and assays: hematology (HumaCount 30, Human, Germany), biochemistry (Humalyzer 3000, Human, Germany), and serology (Elisys Uno, Human, Germany). HIV-testing was done in accordance with the National algorithm, i.e. using a WHO approved rapid test kit (HIV 1 + 2 Antibody Colloidal Gold [KHB], Shanghai Kehua Bio-engineering co., China) for screening, and another rapid test kit (HIV 1/2 STAT-PAK, Chembio Diagnostics, USA) for confirmation.

Testing for hepatitis B e-antigen (HBeAg), anti-HBe, HBV DNA viral load, HBV genotyping and hepatitis delta virus (HDV) was performed at the Norwegian Public Health Institute (Oslo, Norway). HBeAg/anti-HBe were analyzed using an enzyme-linked fluorescent immunoassay (VIDAS HBe/anti-HBe, BioMérieux, Marcy l’Etoile, France). HDV antibodies were detected using an enzyme-linked immunosorbent assay (ETI-AB-DELTAK-2, Diasorin, Italy).

The Abbott RealTime HBV assay (Abbott Molecular, Des Moines, USA) was used for viral load testing, following the manufacturer’s instructions. For genotyping an in-house sequence based assay was used. After extraction with the Abbot sp2000 system (Abbott Molecular, Des Moines, USA), HBV DNA was amplified using HBV-specific primers covering the small S-gene region 5′– GACCCCTGCTCGTGTTA −3` (forwards) and 5′– TGAATACTTTCCAATCAATAGG – 3′ (reverse). The applied PCR conditions were AB-gene PCR-buffer with Syber green (0.33×), Pt-Taq, 3 mM Mg, 0.5 uM primer, 0.2 mM dNTP. The thermal cycling conditions were: denaturation, 95 °C for 2 min; amplification, 45 cycles of 15 s at 95 °C, 45 s at 65 °C, 90 s at 72 °C. The PCR products (797 bp) were further sequenced using Big Dye Terminator v3.1 on the ABI Prism 3100 instrument (Applied Biosystems, Foster City, USA). Sequencing conditions were: 25 cycles at 96 °C for 15 s followed by annealing and elongation at 60 °C for 4 min. The HBV genotype was determined using the genotyping databases (www.geno2pheno.org) at the Max-Planck-Institute for informatics (http://hbv.bioinf.mpi-inf.mpg.de).

### Assessment of liver fibrosis

Liver fibrosis was assessed using transient elastography (Fibroscan 402, Echosense, France) and was part of the baseline examination in all patients. Patients were instructed to fast for at least 2 h prior to the examination, and the procedure was performed by a trained, experienced operator as per the manufacturer’s instructions. The median of 10 readings was employed, and the result was discarded if the interquartile range (IQR) divided by the median exceeded 30%.

A Fibroscan threshold of 7.9 kPa was used to define significant fibrosis (Metavir score ≥ F2), based on a meta-analysis of Asian and European patients [16] and a recent study from West Africa [17]. To define cirrhosis (Metavir score F4), the West African study identified 9.5 kPa as the optimal threshold, which is lower than most other studies. We decided to use a Fibroscan threshold of 9.9 kPa to define cirrhosis, both for simplicity (“10 kPa and above is cirrhosis”) and to improve specificity. In patients with grossly elevated ALT (a “flare”), Fibroscan measurements were repeated over a period of at least 3 months, since liver stiffness can be overestimated in this situation [18]. Ultrasound of the liver was performed in patients who started antiviral treatment, mainly to exclude HCC.

### Treatment eligibility

The present treatment program in Ethiopia was established prior to the release of the WHO guidelines for CHB treatment; hence, we based treatment eligibility criteria in our program on the European Association for the Study of the Liver (EASL) guidelines from 2012, which recommend treatment in the following situations: a) significant fibrosis and viral load >2000 IU/mL, or b) moderate/severe necroinflammation (Metavir score ≥ A2 on liver biopsy) and viral load >2000 IU/mL, or c) alanine aminotransferase (ALT) >80 U/L and viral load >20,000 IU/mL, or d) cirrhosis and any detectable viral load [2]. Some modifications of the EASL criteria were required to fit a low-income setting; specifically, since liver biopsy was unavailable in our setup, we merged criteria b and c into a new one: ALT >80 U/L and viral load >2000 IU/mL. Furthermore, since Africans with CHB are at particular risk of HCC [1], we added a criterion about HCC in near family.

Thus, patients who fulfilled the following criteria were considered eligible for treatment in the present program:

- Decompensated cirrhosis
- Compensated cirrhosis (confirmed with ultrasound or Fibroscan)
- ALT >80 U/L (i.e. 2 times the upper limit of normal) and viral load >2000 IU/mL
- Fibroscan >7.9 kPa and viral load >2000 IU/mL
- HCC among first-grade relative and viral load >2000 IU/mL

Patients who were eligible for treatment were given adherence counseling before starting antiviral treatment. Based on its potency, low risk of resistance, safety profile and favorable costs, TDF (Viread*,* Gilead Sciences Inc., USA) 300 mg once daily was the drug used in the current study.

In a separate analysis, we compared the treatment eligibility criteria employed in the present program (“St. Paul criteria”) with those of the EASL guidelines from 2012 and 2017 and the WHO guidelines from 2015. Treatment experienced patients were excluded from this analysis since the basis for treatment eligibility assessment (mainly ALT and viral load) could be affected by ongoing therapy. The EASL 2017 criteria were the same as the EASL 2012 criteria (a to d) described above, with the addition of: e) HBeAg positive patients older than 30 years of age, and f) a family history of HCC or cirrhosis [19]. The WHO criteria were: a) clinically diagnosed cirrhosis, or b) aspartate aminotransferase to platelet ratio index (APRI) >2.0, or c) age ≥ 30 years with abnormal ALT and viral load >20,000 IU/mL [1].

### Statistical analysis

Descriptive statistics were used to summarize patient demographics and other baseline characteristics. Logistic regression models were used to study associations between baseline variables and the presence of significant liver fibrosis, defined as a Fibroscan value above 7.9 kPa [16]. Six patients with ALT level above 200 U/L (i.e. more than 5 times the upper limit of normal) were excluded from this analysis since ALT elevation can cause falsely elevated Fibroscan results [18]. Univariable logistic regression was computed for the following variables: age, gender, alcohol abuse, HBeAg, HBV genotype, HDV co-infection, ALT level and HBV viral load. Variables with a *p*-value below 0.1 in univariable analyses were included in the multivariable logistic regression model, using a forward stepwise method. Multi-collinearity was excluded using Spearman’s correlation coefficient with a cutoff at 0.7. All tests were two-sided and the significance level was set at *P* < 0.05. Data were analyzed using SPSS for Windows version 23.0 (SPSS Inc., Chicago, USA).

## Results

### Baseline characteristics

Among the initial 300 patients, 138 (46.0%) were women, of whom 19 (13.7%) were pregnant and 16 (12.0%) were breastfeeding. The median age of the study participants was 30 years (interquartile range [IQR] 26–40). Co-infections were relatively rare: 5 patients (1.7%) had HIV-infection and were referred to HIV care, 4 patients (1.3%) had hepatitis C co-infection, and 11 patients (3.7%) were anti-HDV positive. Alcohol abuse was reported in 5 patients (1.7%) and khat abuse in 18 (6.0%). Baseline characteristics of the study participants are summarized in Table 1.

**Table 1:** Baseline characteristics of 300 patients with chronic hepatitis B, Addis Ababa, Ethiopia

Characteristics	Number (%)
Sex	
Male	162 (54.0)
Female	138 (46.0)
Age group (years)	
18–25	65 (21.7)
26–35	129 (43.0)
36–45	60 (20.0)
> 45	46 (15.3)
Marital status	
Single	104 (34.7)
Married	185 (61.7)
Divorced/widowed	11 (3.6)
Previous antiviral treatment	
Yes	29 (9.7)
No	271 (90.3)
Substance abuse	
Alcohol	5 (1.7)
Khat	18 (6.0)
Fasting TE value (kPa) (*n* = 268)	
≤ 7.9	189 (70.5)
8.0–9.9	29 (10.8)
> 9.9	50 (18.7)
ALT (U/L) (*n* = 295)	
≤ 40	245 (83.1)
41–80	34 (11.5)
> 80	16 (5.4)
HBeAg (*n* = 289)	
Positive	27 (9.3)
Negative	262 (90.7)
HBV viral load (IU/ml) (*n* = 294)	
< 2000	169 (57.5)
2000–20,000	60 (20.4)
≥ 20,000	65 (22.1)
HBV genotype (*n* = 79)	
A	67 (84.8)
D	12 (15.2)
Co-infections	
HIV	5 (1.7)
HCV	4 (1.3)
HDV (*n* = 294)	11 (3.7)

*TE* transient elastography, *ALT* alanine aminotransferase, *HBeAg* hepatitis B e-antigen, *HBV* hepatitis B virus, *HIV* human immunodeficiency virus, *HCV* hepatitis C virus, *HDV* hepatitis D virus

Twenty-nine patients (9.7%) reported previous exposure to antiviral treatment, either through the private sector or from the “black market”. The antiviral regimens reported by these patients were: TDF (*n* = 15), TDF/lamivudine fixed-dose combination (*n* = 7), lamivudine (*n* = 3), and pegylated interferon (*n* = 4). By the time of enrolment in the study, 17 were still taking their antiviral drugs, of whom 11 had a completely suppressed viral load (<15 IU/ml). The remaining had quit either for financial reasons or because they could no longer access the drugs or because they had completed the prescribed course of pegylated interferon.

The majority of patients were HBeAg negative (*n* = 262; 90.7%) and had a low viral load: median viral load was 1129 IU/ml (IQR 191–12,861). Similarly, most patients had a normal ALT: median ALT was 23 U/L (IQR 16–33). HBV genotyping was performed in 79 patients, and only genotypes A and D were identified.

Transient elastography was successful in 268 patients, and the median Fibroscan value was 5.8 kPa (IQR 4.8–8.0). Thirty-two patients (10.7%) did not have a valid Fibroscan result; the main reasons for an invalid or missing result were pregnancy (elastography contraindicated, *n* = 14), ascites (elastography not feasible, *n* = 7) and overweight/obesity (body mass index >25 kg/m^2^; *n* = 7).

### Antiviral treatment

Out of all 300 patients in this analysis, 74 (24.7%) met the treatment eligibility criteria and started treatment, the majority of whom (*n* = 46; 62.2%) had cirrhosis. Four patients with cirrhosis did not start treatment; one died shortly after enrolment, one refused treatment, and two had advanced HCC at ultrasonography and were transferred to palliative care. At the other end of the scale, 103 of 265 patients (38.9%) with a complete laboratory profile at baseline, had entirely normal findings (Fibroscan ≤7.9 kPa, viral load <2000 IU/ml, ALT ≤40 U/L), suggesting that they were inactive carriers.

The various treatment guidelines were compared in a subset of 271 treatment naïve patients. As expected, there was a good concordance between the St. Paul criteria and the EASL 2012 criteria (Table 2). The EASL 2017 guidelines introduced two new eligibility criteria; hence, the number of eligible patients was higher compared to the previous version. Of concern, using the WHO 2015 guidelines, only 29 patients (10.7%) were eligible for treatment, most of whom had decompensated cirrhosis.

**Table 2:** Comparison of treatment eligibility criteria among treatment-naïve chronic hepatitis B patients, Ethiopia (*N* = 271)

St. Paul		EASL 2012		EASL 2017		WHO 2015	
*Criteria*	*N*	*Criteria*	*N*	*Criteria*	*N*	*Criteria*	*N*
Decompensated cirrhosis	20	Cirrhosis and detectable VL	40	Cirrhosis and detectable VL	40	Decompensated cirrhosis	20
Compensated cirrhosis	20	Significant fibrosis and VL >2000 IU/ml	8	Significant fibrosis and VL >2000 IU/ml	8	APRI >2.0^a^	4
Significant fibrosis and VL >2000 IU/ml	8	ALT >80 U/L and VL >20,000 IU/ml	1	ALT >80 U/L and VL >20,000 IU/ml	1	Age ≥ 30 years and ALT >40 U/L and VL >20,000 IU/ml	5
ALT >80 U/L and VL >2000 IU/ml	3	Metavir ≥A2 and VL >2000 IU/ml^b^	0	Metavir ≥A2 and VL >2000 IU/ml^b^	0		
Family history HCC and VL >2000 IU/ml	5			HBeAg positive and age ≥ 30 years	3		
				Family history HCC or cirrhosis	16		
Total	56	Total	49	Total	68	Total	29

*ALT* alanine aminotransferase, *VL* viral load, *HCC* hepatocellular carcinoma, *APRI* aspartate aminotransferase to platelet ratio index

^a^Platelet counts were missing and APRI could not be calculated in 20 patients

^b^Liver biopsy was not performed; hence, none fulfilled this criterion

### Factors associated with liver fibrosis

**Fig. 1:**
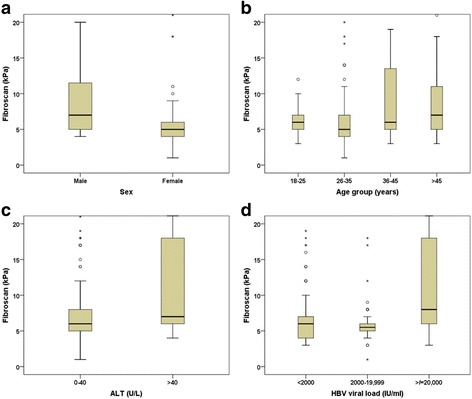
Liver fibrosis as a function of **a** sex, **b** age, **c** ALT and **d** HBV viral load in a cohort of patients with chronic hepatitis B in Ethiopia. Patients with invalid/missing Fibroscan results or ALT elevated more than 5 times the upper limit of normal were excluded from this analysis

Seventy-nine patients had significant liver fibrosis (Fibroscan >7.9 kPa) at baseline. Figure 1 illustrates the degree of fibrosis as a function of age, sex, ALT and viral load level.

Logistic regression analysis, after exclusion of six patients with a grossly elevated ALT, showed that male sex, increasing age, HBeAg positivity, ALT >40 U/L and viral load >20,000 IU/ml were significantly associated with liver fibrosis in univariable analysis. In the multivariable logistic regression model, only sex, age and viral load remained significant (Table 3).

**Table 3:** Baseline variables associated with significant fibrosis (Fibroscan >7.9 kPa) among Ethiopian patients with chronic hepatitis B (*n* = 260)

	No fibrosis N (%)	Significant fibrosis N (%)	Crude		Adjusted	
Baseline variable			OR (95% CI)	*P*	OR (95% CI)	*P*
Gender						
Female	99 (53.2)	17 (23.0)	1		1	
Male	87 (46.8)	57 (77.0)	3.8 (2.1–7.0)	<0.001	3.0 (1.5–5.9)	0.002
Age group (years)						
18–25	41 (22.0)	12 (16.2)	1		1	
26–35	92 (49.5)	23 (31.1)	0.9 (0.4–1.9)	0.695	1.5 (0.6–3.9)	0.406
36–45	30 (16.1)	22 (29.7)	2.5 (1.1–5.8)	0.033	4.4 (1.5–12.8)	0.006
> 45	23 (12.4)	17 (23.0)	2.5 (1.0–6.2)	0.043	3.6 (1.2–11.0)	0.022
Alcohol abuse						
No	184 (98.9)	71 (95.9)	1			
Yes	2 (1.1)	3 (4.1)	3.9 (0.6–23.8)	0.142		
HBeAg (*n* = 255)						
Negative	174 (95.1)	60 (83.3)	1		1	
Positive	9 (4.9)	12 (16.7)	3.9 (1.6–9.6)	0.004	2.7 (0.9–8.4)	0.087
HBV genotype (*n* = 68)						
A	38 (82.6)	19 (86.4)	1			
D	8 (17.4)	3 (13.6)	0.8 (0.2–3.2)	0.695		
HDV co-infection (*n* = 259)						
No	180 (97.3)	72 (97.3)	1			
Yes	5 (2.7)	2 (2.7)	1.0 (0.2–5.3)	1.000		
ALT (U/L)						
≤ 40	166 (89.2)	56 (75.7)	1		1	
> 40	20 (10.8)	18 (24.3)	2.7 (1.3–5.4)	0.006	1.8 (0.8–4.0)	0.162
HBV viral load (IU/ml) (*n* = 259)						
< 2000	114 (61.6)	37 (50.0)	1		1	
2000–19,999	47 (25.4)	9 (12.2)	0.6 (0.3–1.3)	0.198	0.7 (0.3–1.6)	0.350
≥ 20,000	24 (13.0)	28 (37.8)	3.6 (1.9–7.0)	<0.001	3.2 (1.4–7.4)	0.006

*OR* odds ratio, *CI* confidence interval, *HBeAg* hepatitis B e-antigen, *HBV* hepatitis B virus, *HDV* hepatitis D virus, *ALT* alanine aminotransferase

## Discussion

In this study, we describe the initial 300 patients who took part in one of the first and largest treatment programs for CHB in sub-Saharan Africa. Of concern, even though most of the patients were below 40 years of age, many had advanced liver disease at enrolment. Nearly one third had significant liver fibrosis, of whom the majority had cirrhosis. Based on our modified EASL treatment criteria, about one quarter of the patients were in need of antiviral treatment. Since this was the first public sector treatment program in Ethiopia, it is unsurprising that symptomatic patients, desperate for lifesaving medicines, were overrepresented in the early phase. In fact, this resembles the situation in HIV care a decade ago, when new treatment programs were often overwhelmed with patients with advanced disease [20]. This underscores the need to scale up CHB treatment in the country, and make antiviral therapy available to those in need.

The vast majority of patients in our cohort were HBeAg negative. This is in line with previous studies indicating that African CHB patients have a lower rate of HBeAg positivity compared to those in the Far East [21]. This phenomenon is explained, at least partly, by geographical variations in HBV genotypes and mode of transmission [22]. Indeed, over the past few decades, HBeAg negative CHB has become the commonest type of HBV infection in many countries of the world. Patients with HBeAg negative CHB have exacerbations with high viral load, elevated ALT and histologic activity, and have been shown to develop cirrhosis faster than patients with HBeAg positive CHB [23]. Timely antiviral therapy is particularly beneficial in this patient group, as studies have shown that suppression of HBV not only stops the progression of liver disease, but also leads to regression of liver fibrosis and cirrhosis [5, 24]. Furthermore, up to 8 years of treatment with TDF has been shown to be effective, safe and without development of resistance [25].

Approximately one third of our cohort had low viral replication and no evidence of liver disease, and were assumed to be “inactive carriers”. Such patients have an excellent long-term prognosis even in the absence of treatment [26, 27]. In low-income settings, it seems reasonable to focus resources on patients who can be expected to benefit the most from follow-up and treatment. Hence, in an Ethiopian context, we decided that inactive carriers could be discharged from the program after a period of one year, provided that they had repeatedly normal laboratory tests. Certainly, the reduced personnel and laboratory costs in these patients must be balanced against the small risk of reactivation of HBV [28]. The cost-effectiveness of this strategy should be investigated in future studies.

Interestingly, our study revealed a low proportion of co-infections with HIV (1.7%), HCV (1.3%) and HDV (3.7%). Several studies from HIV cohorts have reported much higher co-infection rates. A recent review article reported HIV/HBV co-infection in the range 5.0–8.3% in East Africa and 2.7–35.7% in West Africa [29]. However, the only previous study to report HIV/HBV co-infection in an African HBV cohort found a relatively low prevalence of HIV (3.3%) among HBsAg positive individuals in The Gambia [30], in keeping with our findings. Although HIV and HBV share the same transmission routes, most adults with CHB have been infected in early childhood and would not have any increased risk of acquiring HIV later in life [31]. The low HIV prevalence in our CHB cohort, therefore, is mirroring the HIV prevalence in the general population in Ethiopia, which is estimated to be 1.2% [12]. Similarly, the low proportion with HBV/HCV co-infection in our study is probably a reflection of the overall low prevalence of HCV in Ethiopia [32]. The presence of anti-HDV antibodies in our study was also relatively low, which is in line with a previous study from East Africa; however, available data from the region is scarce [33].

Male sex, age above 35 years and HBV viral load above 20,000 IU/ml were strong and significant predictors of advanced liver disease in our cohort. The association with sex and age observed in our study is similar to reports from elsewhere [34]. Increasing age is thought to be a proxy indicator of longstanding HBV infection, whereas the association between male sex and fibrosis, although consistently reported throughout the world, is less intuitive. High HBV DNA level was shown to be a strong risk factor for development of cirrhosis in the REVEAL study in Taiwan [26], in line with our study. There was no significant association between fibrosis and alcohol abuse, HBeAg status, HDV co-infection, ALT level or genotype in our cohort. In a recent longitudinal study from The Gambia, male sex, maternal HBsAg status, genotype A (compared to E), aflatoxin exposure, HBeAg status, HBV viral load and ALT level were independent predictors of development of fibrosis [35]. Local data from Africa are important to inform evidence-based guidelines, and the present study is one of few to report predictors of liver fibrosis in CHB mono-infected patients on the continent.

Our study had certain limitations. First, we used Fibroscan for staging of liver fibrosis, and since the threshold to identify cirrhosis (9.9 kPa) was lower than in many other studies, it is possible that we overestimated the prevalence of cirrhosis. However, in the largest study on the African continent a Fibroscan threshold of 9.5 kPa yielded a sensitivity of 100% and specificity of 89% [17]. Second, predictors of fibrosis were investigated using a cross-sectional design, and thus the direction of any association cannot be ascertained. On the other hand, the risk factors identified in our study were well in line with studies from elsewhere, and we believe the results are valid.

## Conclusion

In summary, among the initial 300 patients in one of the first and largest CHB treatment programs in sub-Saharan Africa, almost one third had significant liver fibrosis at enrolment. Male sex, age above 35 years and viral load above 20,000 IU/ml were strong and significant predictors of liver fibrosis. Although the vast majority were HBeAg negative and had normal ALT, approximately one quarter of the patients were in need of antiviral treatment, most of whom had cirrhosis. Òur study describes early experiences from establishing CHB care and treatment in a resource-limited setting, and we believe our findings can inform national and international efforts to scale up treatment globally.

## Abbreviations

ALT: Alanine aminotransferase
AST: Aspartate aminotransferase
CHB: Chronic hepatitis B
CI: Confidence interval
DNA: Deoxyribonucleid acid
EASL: European Association for the Study of the Liver
HBeAg: Hepatitis B e-antigen
HBsAg: Hepatitis B surface antigen
HBV: Hepatitis B virus
HCC: Hepatocellular carcinoma
HCV: Hepatitis C virus
HDV: Hepatitis D virus
HIV: Human immunodeficiency virus
IQR: Interquartile range
OR: Odds ratio
TDF: Tenofovir disoproxil fumarate
TE: Transient elastography
WHO: World Health Organization

## References

[1] World Health Organization (2015). Guidelines for the prevention, care and treatment of persons with chronic hepatitis B infection.

[2] European Association for the Study of the Liver (2012). EASL clinical practice guidelines: management of chronic hepatitis B virus infection. J Hepatol.

[3] World Health Organization. Global health sector strategy on viral hepatitis. Towards ending viral hepatitis. Geneva: WHO; 2016.

[4] Pol S, Lampertico P (2012). First-line treatment of chronic hepatitis B with entecavir or tenofovir in 'real-life' settings: from clinical trials to clinical practice. J Viral Hepat.

[5] Marcellin P, Gane E, Buti M (2013). Regression of cirrhosis during treatment with tenofovir disoproxil fumarate for chronic hepatitis B: a 5-year open-label follow-up study. Lancet.

[6] Kim WR, Loomba R, Berg T (2015). Impact of long-term tenofovir disoproxil fumarate on incidence of hepatocellular carcinoma in patients with chronic hepatitis B. Cancer.

[7] Terrault NA, Bzowej NH, Chang KM (2016). AASLD guidelines for treatment of chronic hepatitis B. Hepatology.

[8] Su TH, Hu TH, Chen CY (2016). Four-year entecavir therapy reduces hepatocellular carcinoma, cirrhotic events and mortality in chronic hepatitis B patients. Liver Int.

[9] Lok AS, Trinh H, Carosi G (2012). Efficacy of entecavir with or without tenofovir disoproxil fumarate for nucleos(t)ide-naive patients with chronic hepatitis B. Gastroenterology.

[10] Nwokediuko SC (2011). Chronic hepatitis B: management challenges in resource-poor countries. Hepat Mon.

[11] World Health Organization (2017). Global hepatitis report, 2017.

[12] World Health Organization. Ethiopia. Factsheets of health statistics*.* Regional office for Africa: WHO; 2016: www.aho.afro.who.int/profiles_information/images/d/d5/Ethiopia-Statistical_Factsheet.pdf. Accessed 5 April 2017.

[13] Belyhun Y, Maier M, Mulu A, Diro E, Liebert UG (2016). Hepatitis viruses in Ethiopia: a systematic review and meta-analysis. BMC Infect Dis.

[14] Ministry of Health (2016). National guideline for prevention and control of viral hepatitis in Ethiopia.

[15] Schiff E, Sorrell M, Maddrey W (2007). Schiff's diseases of the liver.

[16] Chon YE, Choi EH, Song KJ (2012). Performance of transient elastography for the staging of liver fibrosis in patients with chronic hepatitis B: a meta-analysis. PLoS One.

[17] Lemoine M, Shimakawa Y, Nayagam S (2016). The gamma-glutamyl transpeptidase to platelet ratio (GPR) predicts significant liver fibrosis and cirrhosis in patients with chronic HBV infection in West Africa. Gut.

[18] Chan HL, Wong GL, Choi PC (2009). Alanine aminotransferase-based algorithms of liver stiffness measurement by transient elastography (Fibroscan) for liver fibrosis in chronic hepatitis B. J Viral Hepat.

[19] European Association for the Study of the Liver. EASL 2017 Clinical practice guidelines on the management of hepatitis B virus infection. J Hepatol 2017; doi:10.1016/j.jhep.2017.03.021. 10.1016/j.jhep.2017.03.021 28427875

[20] Johannessen A, Naman E, Ngowi BJ (2008). Predictors of mortality in HIV-infected patients starting antiretroviral therapy in a rural hospital in Tanzania. BMC Infect Dis.

[21] Kiire CF (1996). The epidemiology and prophylaxis of hepatitis B in sub-Saharan Africa: a view from tropical and subtropical Africa. Gut.

[22] Kramvis A (2016). The clinical implications of hepatitis B virus genotypes and HBeAg in pediatrics. Rev Med Virol.

[23] Alexopoulou A, Karayiannis P (2014). HBeAg negative variants and their role in the natural history of chronic hepatitis B virus infection. World J Gastroenterol.

[24] Buti M, Fung S, Gane E (2015). Long-term clinical outcomes in cirrhotic chronic hepatitis B patients treated with tenofovir disoproxil fumarate for up to 5 years. Hepatol Int.

[25] Liu Y, Corsa AC, Buti M (2017). No detectable resistance to tenofovir disoproxil fumarate in HBeAg+ and HBeAg- patients with chronic hepatitis B after 8 years of treatment. J Viral Hepat.

[26] Iloeje UH, Yang HI, Su J (2006). Predicting cirrhosis risk based on the level of circulating hepatitis B viral load. Gastroenterology.

[27] Chen CJ, Yang HI, Su J (2006). Risk of hepatocellular carcinoma across a biological gradient of serum hepatitis B virus DNA level. JAMA.

[28] Sharma SK, Saini N, Chwla Y (2005). Hepatitis B virus: inactive carriers. Virol J.

[29] Matthews PC, Geretti AM, Goulder PJ, Klenerman P (2014). Epidemiology and impact of HIV coinfection with hepatitis B and hepatitis C viruses in sub-Saharan Africa. J Clin Virol.

[30] Lemoine M, Shimakawa Y, Njie R (2016). Acceptability and feasibility of a screen-and-treat programme for hepatitis B virus infection in the Gambia: the prevention of liver fibrosis and cancer in Africa (PROLIFICA) study. Lancet Glob Health.

[31] Kim WR (2009). Epidemiology of hepatitis B in the United States. Hepatology.

[32] Hundie GB, Raj VS, GebreMichael D, Haagmans BL (2017). Seroepidemiology of hepatitis B and C virus infections among blood donors in Ethiopia. J Med Virol.

[33] Winter A, Letang E, Vedastus Kalinjuma A (2016). Absence of hepatitis delta infection in a large rural HIV cohort in Tanzania. Int J Infect Dis.

[34] Fung J, Lai CL, But D, Wong D, Cheung TK, Yuen MF (2008). Prevalence of fibrosis and cirrhosis in chronic hepatitis B: implications for treatment and management. Am J Gastroenterol.

[35] Shimakawa Y, Lemoine M, Njai HF, et al. Natural history of chronic HBV infection in West Africa: a longitudinal population-based study from the Gambia. Gut. 2016;65:2007–16. 10.1136/gutjnl-2015-309892 26185161

